# Inhibitory Effect of Yongdamsagan-Tang Water Extract, a Traditional Herbal Formula, on Testosterone-Induced Benign Prostatic Hyperplasia in Rats

**DOI:** 10.1155/2016/1428923

**Published:** 2016-07-18

**Authors:** Eunsook Park, Mee-Young Lee, Woo-Young Jeon, Nari Lee, Chang-Seob Seo, Hyeun-Kyoo Shin

**Affiliations:** K-herb Research Center, Korea Institute of Oriental Medicine, 1672 Yuseong-daero, Yuseong-gu, Daejeon 34054, Republic of Korea

## Abstract

Yongdamsagan-tang, a traditional herbal formula, is used widely for the treatment of inflammation and viral diseases. In this study, we investigated whether Yongdamsagan-tang water extract (YSTE) affects testosterone propionate- (TP-) induced benign prostatic hyperplasia (BPH) in a rat model. To induce BPH, rats were injected subcutaneously with 10 mg/kg of TP every day. YSTE was administrated daily by oral gavage at doses of 200 and 500 mg/kg along with the TP injection. After 4 weeks, prostates were collected, weighed, and analyzed. The relative prostrate weight was significantly lower in both YSTE groups (200 and 500 mg/kg/day) compared with the TP-induced BPH group. YSTE administration reduced the expression of proliferation markers PCNA, cyclin D1, and Ki-67 and the histological abnormalities observed in the prostate in TP-induced BPH rats. YSTE attenuated the increase in the TP-induced androgen concentration in the prostate. The YSTE groups also showed decreased lipid peroxidation and increased glutathione reductase activity in the prostate. These findings suggest that YSTE effectively prevented the development of TP-induced BPH in rats through antiproliferative and antioxidative activities and might be useful in the clinical treatment of BPH.

## 1. Introduction

Benign prostate hyperplasia (BPH) is characterized by nonmalignant enlargement of the prostate and is one of the most common urological diseases in elderly men [1, 2]. BPH involves increases in the numbers of both stromal and epithelial cells in the transitional zone of the prostate and can cause lower urinary tract symptoms (LUTS) including urgency, urge incontinence, frequency, nocturia, dysuria, and suprapubic pain [3, 4].

Although the pathogenesis of BPH has not been defined clearly, the relationship between the concentrations of androgens (male sex hormones) and BPH in the aging male population is well known. The serum concentrations of testosterone and dihydrotestosterone (DHT) decrease with age in healthy men. Unlike testosterone, serum DHT level is significantly higher in BPH patients than in healthy men at the same age [5]. A cross-sectional study of 505 men aged 40–79 years reported that increases in the serum DHT level and DHT/testosterone ratio were related to larger prostate volume and higher incidence of BPH [6]. DHT, a metabolite of testosterone, is converted from testosterone in the prostate by 5*α*-reductase enzymes and is a critical mediator of prostate growth [7]. Given the importance of DHT in the development of BPH, inhibitors of 5*α*-reductase, such as finasteride and dutasteride, are used in the clinical treatment of BPH. Inhibition of 5*α*-reductase by these drugs prevents the conversion of testosterone to DHT and reduces DHT level and thereby suppresses hyperplastic growth of the prostate [8]. However, these drugs also have side effects on the nervous system (e.g., decreased libido) and on the urogenital system (e.g., erectile dysfunction, dysuria, and ejaculatory disorders) [9].

Yongdamsagan-tang (YST), a traditional herbal formula, which is also known as Long Dan Xie Gan Tang in Chinese and Ryutanshakan-to in Japanese, contains the extracts of herbs such as Gentian Scabrae Radix, Bupleuri Radix, Alismatis Rhizoma, Akebiae Caulis, Plantaginis Semen, Poria Sclerotium, Rehmanniae Radix Crudus, Angelicae Gigantis Radix, Gardeniae Fructus, Scutellariae Radix, and Glycyrrhizae Radix [10]. Traditionally, YST has been used in the treatment of inflammation and viral diseases such as acute conjunctivitis, acute otitis media, acute hepatitis, acute cholecystitis, acute pyelonephritis, cystitis, and orchitis [10]. Recently, it has been reported that YST inhibits the synthesis of nitric oxide and cell proliferation* in vitro* [11, 12]. In immune dysfunction induced mice model, YST has immunomodulatory effects with the reduction of oxidative stress [13]. In a polycystic ovary rat model, YST administration reduced the number of cystic follicles and the expression of nerve growth factor in the ovaries [14]. Moreover, clinical data have revealed that the treatment of YST in patients with chronic prostatitis causes improvement in the quality of life of patients [15]. Although BPH development is also known to be associated with proliferation and oxidative stress [16, 17], the pharmacological effect of YST against BPH has yet been established. In this study, we investigated whether Yongdamsagan-tang water extract (YSTE) has therapeutic efficacy against BPH in a testosterone propionate- (TP-) induced BPH rat model.

## 2. Materials and Methods

### 2.1. Plant Materials

The 11 raw herbal medicines of YST (Table 1) were purchased from Kwangmyungdag Medicinal Herbs (Ulsan, Korea) in February 2012. Each herbal material was identified by Professor Je Hyun Lee, Department of Herbology, College of Oriental Medicine, Dongguk University, Gyeongju, Korea, and Professor Young-Bae Seo, Department of Herbology, College of Oriental Medicine, Daejeon University, Daejeon, Korea. A voucher specimen (2012-KE49-1~KE49-11) has been deposited at the K-herb Research Center, Korea Institute of Oriental Medicine.

### 2.2. Preparation of YSTE

Preparation of herbal decoction was performed as previously described [18]. Namely, to prepare the YST decoction, the raw materials, 714.3 g of Gentiana Scabra Radix, 714.3 g of Bupleuri Radix, 714.3 g of Alismatis Rhizoma, 357.1 g of Akebia Caulis, 357.1 g of Plantaginis Semen, 357.1 g of Poria Sclerotium, 357.1 g of Rehmanniae Radix Crudus, 357.1 g of Angelicae Gigantis Radix, 357.1 g of Gardeniae Fructus, 357.1 g of Scutellariae Radix, and 357.1 g of Glycyrrhizae Radix et Rhizoma, were mixed. Extraction was performed using 50 L of distilled water at 100°C for 2 hours under pressure (98 kPa) using an electric extractor (COSMOS-660, Kyungseo Machine Co., Incheon, Korea). After extraction, the solution was filtered using a standard sieve (number 270, 53 *μ*m, Chung Gye Sang Gong Sa, Seoul, Korea). The filtered solution was freeze-dried in a freeze drier (PVTFD10RS, Ilshin BioBase, Yangju, Korea) and produced 926.4 g of a powder of lyophilized YSTE (yield: 18.5%).

### 2.3. Animals

Specific Pathogen Free (SPF) seven-week-old male Sprague-Dawley rats weighing 200–220 g were obtained from Orient Bio Inc. (Seoul, Korea) and were maintained in animal facility under a 12-hour light/dark cycle at 18–23°C and 40–60% relative humidity. All rats were fed standard laboratory chow (Harlan Teklad, Madison, WI) and allowed access to water* ad libitum*. All experimental procedures were performed in accordance with the National Institute of Health Guidelines for the Care and Use of Laboratory Animals and were approved by the Korea Institute of Oriental Medicine Institutional Animal Care and Use Committee. Animals were cared for in accordance with the dictates of the National Animal Welfare Law of Korea.

### 2.4. Experimental Design

Induction of BPH in the rat model and experimental procedures were performed as previously described [18]. Rats were randomly assigned to five groups (*n* = 5 per group) and treated for 4 weeks as follows. The first group was the negative control (NC) group, which was given daily phosphate-buffered saline (PBS) orally and subcutaneous (s.c.) injection of corn oil. The second group was the BPH group, which was given daily PBS orally and s.c. injection of 10 mg/kg of TP (Tokyo Chemical Industry Co., Tokyo, Japan) dissolved in corn oil. The third group was the positive control group, which was given daily 10 mg/kg of finasteride (Sigma-Aldrich, St Louis, MO) orally and s.c. injection of 10 mg/kg of TP. The fourth and fifth groups were given daily 200 and 500 mg/kg of YSTE orally and s.c. injection of 10 mg/kg of TP. Oral administration and s.c. injection were conducted sequentially from first to fifth group.

Following the final injection and overnight fasting, the rats were anesthetized with an intraperitoneal injection of 50 mg/kg of pentobarbital. Blood samples were collected, and prostate tissues were immediately dissected and weighed. Sections of prostate lobes were fixed with 4% paraformaldehyde for histological analysis, and the remaining prostate sections were stored at −80°C for further analysis.

### 2.5. Measurement of Testosterone and DHT Levels in the Prostate

Prostate lysates were prepared as previously described [18]. The concentrations of testosterone and DHT in prostate lysates were measured using an enzyme-linked immunosorbent assay (ELISA) kit (ALPCO Diagnostics, Salem, NH) according to the manufacturer's instructions. The absorbance was measured at 450 nm using a microplate reader (Bio-Rad Laboratories Inc., Hercules, CA).

### 2.6. Histological Examination and Immunohistochemistry

Fixed prostate tissues were embedded in paraffin wax and cut into 5 *μ*m thick sections. The sections were deparaffinized, rehydrated, and stained with Mayer's hematoxylin (MHS-16, Sigma-Aldrich) and eosin (HT110-1-32, Sigma-Aldrich) (H&E) solution using standard procedures. The sections were mounted with mounting medium (Invitrogen, Carlsbad, CA) and observed under light microscopy with bright-field illumination (Olympus, Tokyo, Japan).

Immunohistochemistry was performed using a Vectastain Elite ABC Kit (Vector Laboratories Inc., Burlingame, CA) according to the manufacturer's instructions. After antigen retrieval processing, the sections were blocked in normal serum and then incubated with anti-Ki-67 (ab16667, Abcam, Cambridge, UK) antibody overnight at 4°C. On the next day, the sections were incubated in biotinylated-secondary antibody and developed using a DAB peroxidase substrate kit (Vector Laboratories) until a signal was seen.

### 2.7. Western Blot Analysis

Western blot analysis was conducted as previously described [18]. Briefly, total proteins were separated by sodium dodecyl sulfate-polyacrylamide gel electrophoresis and transferred to a polyvinylidene difluoride membrane. The signals were visualized using SuperSignal West Femto Maximum Substrate System (Thermo Scientific, Rockford, IL) and then detected using a ChemiDoc*™* XRS imaging system (Bio-Rad Laboratories). The following antibodies were used: antiproliferating cell nuclear antigen (PCNA) (ab29, Abcam), anti-cyclin D1 (ab134175, Abcam), and anti-*β*-actin (#4967, Cell Signaling, Danvers, MA).

### 2.8. Measurement of Malondialdehyde (MDA) Concentration and Glutathione Reductase (GR) Activity

The concentration of MDA in the prostate was measured by using a QuantiChrom TBARS assay kit (Bioassay Systems, Hayward, CA) according to the manufacturer's instructions. The absorbance was measured at 535 nm, and MDA values are expressed as nmol MDA/mg protein.

GR activity in the prostate was analyzed by using a Glutathione Reductase Assay Kit (Cayman Chemical Co., Ann Arbor, MI) according to the manufacturer's instructions. The absorbance was measured at 340 nm, and GR activity is expressed as units/mg protein.

### 2.9. Statistical Analysis

The data are presented as mean ± standard deviation (SD). Statistical significance was calculated using one-way analysis of variance (ANOVA) with Dunnett's test [19]. Differences were considered significant at *P* < 0.05.

## 3. Results

### 3.1. YSTE Represses Prostate Enlargement in BPH Rats

**Figure 1:**
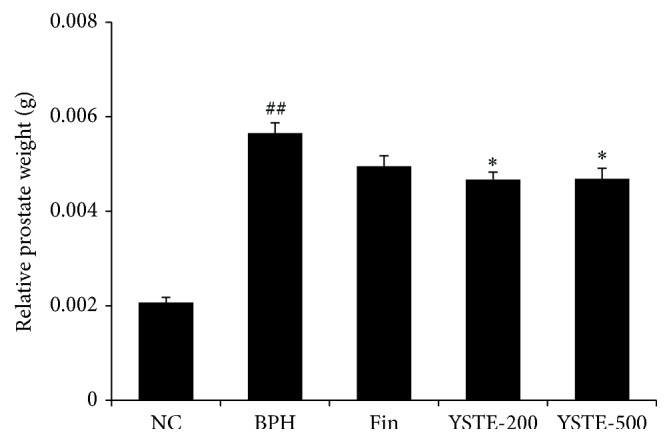
Effect of YSTE on prostate weight. Relative prostate weight was evaluated by normalization to body weight (organ wt/body wt). Values are presented as mean ± SD (*n* = 5). ^##^ *P* < 0.01, compared with the NC group; ^*∗*^ *P* < 0.05, compared with the BPH group. NC, negative control; BPH, TP-induced BPH; Fin, administration of finasteride and s.c. injection of TP; YSTE-200, administration of 200 mg/kg/day of YSTE and s.c. injection of TP; YSTE-500, administration of 500 mg/kg/day of YSTE and s.c. injection of TP.

To determine the effects of YSTE on prostate growth, we measured the changes in prostate weight caused by TP and any effects of YSTE. The relative prostate weight was higher in the TP-induced BPH group compared with the NC group. The relative prostate weight was significantly lower in the 200 and 500 mg/kg/day-treated YSTE groups and finasteride-treated group compared with the TP-induced BPH group (Figure 1). In contrast to the prostate weights, at the end of the study, the body weights were similar in all groups (data not shown). Interestingly, the effect of YSTE on prostate growth was independent of dosage.

**Figure 2:**
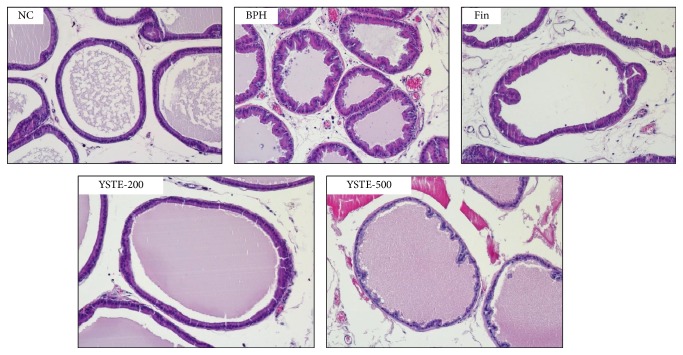
Effect of YSTE on histomorphology of the prostate in BPH rats. Representative H&E histology of prostate sections from BPH rats (magnification, ×100). NC, negative control; BPH, TP-induced BPH; Fin, administration of finasteride and s.c. injection of TP; YSTE-200, administration of 200 mg/kg/day of YSTE and s.c. injection of TP; YSTE-500, administration of 500 mg/kg/day of YSTE and s.c. injection of TP.

To examine whether YSTE affects the histological abnormalities associated with BPH, prostate samples were stained with H&E. As shown in Figure 2, the prostates in the TP-induced BPH group exhibited prostatic hyperplasia with multiple layers of epithelial cells. This contrasted with the prostates in the NC group, which exhibited epithelial cells arranged in a single layer. The histological abnormalities associated with BPH were reduced substantially in the groups given YSTE and finasteride. These data suggest that the reduced prostate weight after YSTE administration resulted from the attenuation of the abnormal histological effects in the prostate of BPH rats.

### 3.2. YSTE Reduces Testosterone and DHT Concentrations in the Prostate of BPH Rats

**Figure 3:**
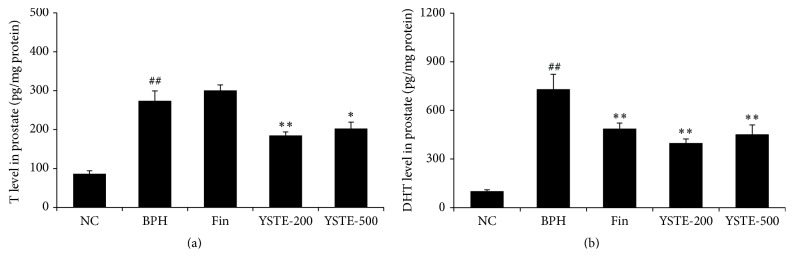
Effect of YSTE on testosterone and DHT concentrations in the prostate. Testosterone (a) and DHT (b) concentration were measured in prostate homogenates. Values are presented as mean ± SD (*n* = 5). ^##^ *P* < 0.01, compared with the NC group; ^*∗*^ *P* < 0.05 and ^*∗∗*^ *P* < 0.01, compared with the BPH group. NC, negative control; BPH, TP-induced BPH; Fin, administration of finasteride and s.c. injection of TP; YSTE-200, administration of 200 mg/kg/day of YSTE and s.c. injection of TP; YSTE-500, administration of 500 mg/kg/day of YSTE and s.c. injection of TP.

Finasteride is a novel therapeutic agent that is known to reduce DHT concentration and prostate size through the selective inhibition of 5-*α*-reductase activity [20]. The concentrations of testosterone and DHT in the prostate were measured to determine whether YSTE also affects androgen levels. As shown in Figures 3(a) and 3(b), testosterone and DHT concentrations were higher in the prostate of the TP-induced BPH group compared with the NC group. Compared with the TP-induced BPH group, the finasteride-treated group had a lower prostate concentration of DHT but not of testosterone, whereas the YSTE-treated groups had lower concentrations of both testosterone and DHT. These data suggest that YSTE administration reduced the androgen concentrations in the prostate of BPH rats.

### 3.3. YSTE Has Antiproliferative Effects in the Prostate of BPH Rats

**Figure 4:**
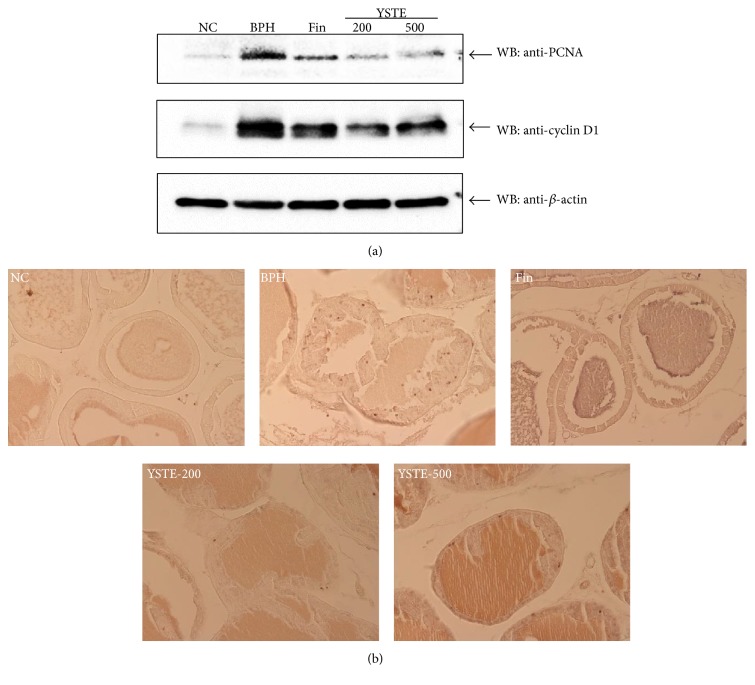
Effect of YSTE on cell proliferation in the prostate in BPH rats. (a) Total prostate protein (30 *μ*g) was subjected to western blot analysis for identification of PCNA and cyclin D1 protein levels. The *β*-actin protein concentration was used as an internal control. (b) The number of Ki-67-positive cells in the prostate from BPH rats was determined by immunohistochemistry using anti-Ki-67 (magnification, ×200). NC, negative control; BPH, TP-induced BPH; Fin, administration of finasteride and s.c. injection of TP; YSTE-200, administration of 200 mg/kg/day of YSTE and s.c. injection of TP; YSTE-500, administration of 500 mg/kg/day of YSTE and s.c. injection of TP.

To elucidate whether YSTE suppresses prostate cell proliferation in BPH rats, we examined the expression of proliferation markers such as PCNA, cyclin D1, and Ki-67. Western blot analysis showed that, compared with the NC group, the levels of PCNA and cyclin D1 protein were markedly higher in the TP-induced BPH group and markedly reduced in the YSTE-treated groups and the finasteride-treated group (Figure 4(a)). Consistent with the expression of PCNA and cyclin D1, an increased number of Ki-67-positive cells was observed in the prostates of the TP-induced BPH group compared with the NC group; this effect was considerably reduced in both YSTE-treated groups and the finasteride-treated group (Figure 4(b)). These data suggest that YSTE administration attenuated BPH through antiproliferative activity.

### 3.4. YSTE Inhibits Oxidative Stress in the Prostate of BPH Rats

**Figure 5:**
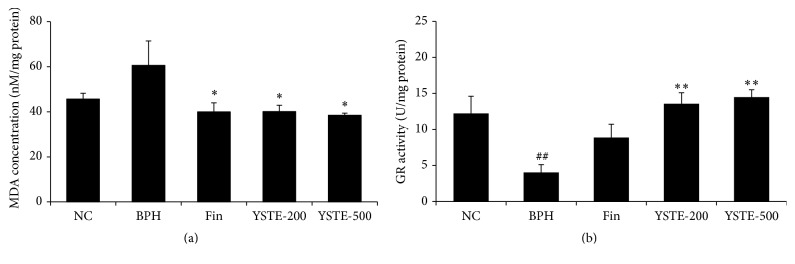
Effect of YSTE on oxidative stress in the prostate of BPH rats. MDA concentration (a) and GR activity (b) were measured in prostate homogenates. Values are presented as mean ± SD (*n* = 5). ^##^ *P* < 0.01, compared with the NC group; ^*∗*^ *P* < 0.05 and ^*∗∗*^ *P* < 0.01, compared with the BPH group. NC, negative control; BPH, TP-induced BPH; Fin, administration of finasteride and s.c. injection of TP; YSTE-200, administration of 200 mg/kg/day of YSTE and s.c. injection of TP; YSTE-500, administration of 500 mg/kg/day of YSTE and s.c. injection of TP.

To investigate the effect of YSTE on oxidative stress in BPH, the concentration of MDA, an indicator of lipid peroxidation, and the activity of GR, an antioxidant enzyme, were measured in the prostate. As shown in Figure 5(a), MDA concentration was significantly higher in the TP-induced BPH group compared with the NC group, as previously reported [21]. By contrast, MDA concentration was significantly reduced in the 200 and 500 mg/kg/day-treated YSTE groups and the finasteride-treated group compared with the TP-induced BPH group. As shown in Figure 5(b), GR activity was significantly lower in the TP-induced BPH group compared with the NC group but was significantly higher in the 200 and 500 mg/kg/day-treated YSTE groups and the finasteride-treated group compared with the TP-induced BPH group. These data suggest that YSTE administration prevented BPH by protecting the prostate from oxidative stress.

## 4. Discussion

BPH is the most common urological disease in men over 50 years old and is characterized by the enlargement of and histological changes in the prostate gland [2]. Given the many side effects of surgery and pharmacological therapy and the long latency of BPH, phytotherapy based on products derived naturally from plants has emerged as an alternative treatment for BPH because it is thought to be less toxic [22]. Therefore, we evaluated the effect of YSTE on TP-induced BPH rat model. YSTE, a traditional herbal formula, administration in TP-induced BPH rats for 4 weeks also showed an inhibitory effect on the proliferation and oxidative stress of BPH as well as pathophysiological feature of BPH such as prostate weight.

In previous studies using rat models, changes in prostate weight and histomorphology have provided the main evidence for the inhibitory effects of substances on BPH development [18, 23, 24]. In the present study, YSTE reduced the relative prostate weight and histological abnormalities in TP-induced BPH rats, which supports the idea that YSTE inhibits BPH development. However, the effect of YSTE on prostate growth was independent of dosage. Thus, YSTE at a dose of 200 mg/kg/day seemed to achieve maximum efficacy in this BPH rat model. Unlike finasteride, YSTE significantly reduced the concentrations of both testosterone and DHT compared with TP treatment. A previous study has shown that gonadectomy, which eliminates testicular testosterone, during the early phase of BPH is more effective in inhibiting prostatic overgrowth than are antiandrogens or 5*α*-reductase inhibitors [25]. Therefore, we suggest that the inhibitory effect of YSTE occurs at the time of the development of BPH.

We also evaluated the mechanism responsible for the inhibitory effects of YSTE on BPH development. The development and progression of BPH are associated with various processes. Uncontrolled proliferation in the prostate increases the number of stromal and epithelial cells, which leads to increased prostate volume [16]. Aberrant cell proliferation is associated with deregulation of the cell cycle, and the expression of cell cycle-regulated proteins has been evaluated to understand how the proliferation is related to various diseases and cancers. Of the cell cycle-regulated proteins, PCNA and cyclin D1 are expressed in G1/S phase, and Ki-67, a proliferation-associated nuclear antigen, is expressed in all phases of the cell cycle except for G0 phase [26]. The expression of PCNA and Ki-67 is increased significantly in patients with BPH or prostate cancer, and the degree of proliferation is related to the clinical grade of prostate cancer [27]. The expression of these proteins in the prostate of BPH animal model is regulated by substances that affect the BPH development [24, 28]. Consistent with previous reports, our study showed that YSTE administration significantly reduced the PCNA and Ki-67 protein levels and the number of Ki-67-positive cells in a TP-induced BPH rat model. These findings suggest that the effects of YSTE on BPH development involve antiproliferative activity.

Various studies have reported an association between oxidative stress and the development of BPH. In a transgenic mouse model in which oxidative DNA damage was induced by the overexpression of Nox gene in the prostatic epithelium, the pathogenesis of BPH involved an increase in prostate weight and epithelial proliferation [29]. BPH patients exhibit increased lipid peroxidation as a result of oxidative stress and decreased antioxidant enzyme activities [17, 30]. In this study, YSTE administration decreased the concentration of MDA, an indicator of lipid peroxidation, and increased the activity of GR, an antioxidant enzyme. Similar to YSTE, several other herbal extracts including* Abacopteris penangiana* and* Melandrium firmum*, have been shown to reduce oxidative stress in a BPH rat model [21, 31]. Taken together, these findings suggest that the inhibitory effects of YSTE on BPH involve its antioxidative activity.

## 5. Conclusion

YSTE administration significantly reduced the relative weight and histological abnormalities of the prostate in TP-induced BPH rats. YSTE administration also reduced the expression of PCNA, cyclin D1, and Ki-67 protein and testosterone and DHT concentrations in TP-induced BPH rats. Moreover, YSTE administration decreased MDA concentration and increased GR activity in TP-induced BPH rats. These findings suggest that YSTE inhibits the development of BPH through antiproliferative and antioxidative activities and might be useful in the clinical treatment of BPH.

## Figures and Tables

**Table 1:** Composition of YST.

Latin name	Scientific name	Ratio (%)	Origin
Gentiana Scabra Radix	*Gentiana scabra *Bunge	2	China
Bupleuri Radix	*Bupleurum falcatum *Linne	2	Gurye, Korea
Alismatis Rhizoma	*Alisma orientale *Juzepczuk	2	Yeongcheon, Korea
Akebia Caulis	*Akebia quinata *Decaisne	1	Yeongcheon, Korea
Plantaginis Semen	*Plantago asiatica *Linne	1	China
Poria Sclerotium	*Poria cocos *Wolf	1	Pyeongchang, Korea
Rehmanniae Radix Crudus	*Rehmannia glutinosa *(Gaertner) Liboschitz ex Steudel	1	Gunwi, Korea
Angelicae Gigantis Radix	*Angelica gigas *Nakai	1	Bonghwa, Korea
Gardeniae Fructus	*Gardenia jasminoides *Ellis	1	Gurye, Korea
Scutellariae Radix	*Scutellaria baicalensis* Georgi	1	Gurye, Korea
Glycyrrhizae Radix et Rhizoma	*Glycyrrhiza uralensis* Fischer	1	China
